# Mechanisms of action of *Lycium barbarum* polysaccharide in protecting against vitiligo mice through modulation of the STAT3-Hsp70-CXCL9/CXCL10 pathway

**DOI:** 10.1080/13880209.2022.2163406

**Published:** 2023-01-18

**Authors:** Liqian Peng, Yue Lu, Yingming Gu, Bihua Liang, Yanhong Li, Huaping Li, Yanan Ke, Huilan Zhu, Zhenjie Li

**Affiliations:** a Department of Dermatology, Guangzhou Institute of Dermatology, Guangzhou, China; b Institute of Dermatology, Guangzhou Medical University, Guangzhou, China; c The Second Affiliated Hospital, Guangzhou University of Chinese Medicine (Guangdong Provincial Hospital of Chinese Medicine), Guangzhou, China

**Keywords:** Depigmentation, CD8+ T cell, immunoregulation, inflammation

## Abstract

**Context:**

Vitiligo is a common skin disease with a complex pathogenesis, and so far, no effective treatment is available. *Lycium barbarum* L. (Solanaceae) polysaccharide (LBP), the main active ingredient of goji berries, has been demonstrated to protect keratinocytes and fibroblasts against oxidative stress.

**Objective:**

This study explored the effects and mechanism of LBP on monobenzone-induced vitiligo in mice.

**Materials and methods:**

C57BL/6 mice were randomly divided into five groups (*n* = 6): negative control that received vaseline, vitiligo model group induced by monobenzone that treated with vaseline, positive control that received tacrolimus (TAC), LBP groups that received 0.3 and 0.6 g/kg LBP, respectively. We quantified the depigmentation by visual examination and scores, detected the expression of CD8+ T cells, pro-inflammatory cytokines and analysed the STAT3-Hsp70-CXCL9/CXCL10 pathway.

**Results:**

LBP 0.3 and 0.6 g/kg groups can significantly reduce depigmentation scores and the infiltration of local inflammatory cells in the skin lesions. Moreover, the expression of CXCL9, CXCL3, CXCL10 and HSP70 decreased by 54.3, 20.3, 48.5 and 27.2% in 0.3 g/kg LBP group, which decreased by 62.1, 26.6, 58.2 and 34.5% in 0.6 g/kg LBP group. In addition, 0.3 and 0.6 g/kg LBP decreased the release of IL-8 (9.7%, 22.8%), IL-6 (40.8%, 42.5%), TNF-α (25.7%, 35%), IFN-γ (25.1%, 27.6%) and IL-1β (23.7%, 33.7%) and inhibited the phosphorylation expression of STAT3 by 63.2 and 67.9%, respectively.

**Conclusion:**

These findings indicated LBP might be recommended as a new approach for vitiligo which provide a theoretical basis for the clinical application of LBP in treating vitiligo patients.

## Introduction

Vitiligo is a chronic acquired autoimmune skin disease that is commonly diagnosed in clinical practice. Vitiligo lesions occur as well-defined depigmented patches on the skin and mucosae, which may be distributed throughout the body; however, lesions typically occur in the perioral and periocular regions, on fingers and arches, and may induce whitening of hair (Taieb et al. 2013). The current global incidence of this disease is approximately 0.5–1%, with 0.1–2% in children (Sehgal and Srivastava 2007; Al-Refu 2012). The treatment of vitiligo is considerably challenging due to typically short remission periods, rapid recurrence and high treatment resistance; most commonly used drugs may cause considerable toxic side effects. Therefore, safe and effective natural remedies are urgently required. Tacrolimus is a classic immunosuppressive drug, which is commonly used in the treatment of vitiligo and was thus chosen as the positive drug in this study (Mumtaz et al. 2020).

A substantial body of clinical evidence suggests that vitiligo is associated with autoimmunity. Patients with vitiligo frequently also suffer from autoimmune thyroid diseases, type I diabetes, malignant anaemia and systemic lupus erythematosus, among others (Ezzedine et al. 2015; Boniface et al. 2018). CD8+ cytotoxic T lymphocytes (CTLs), as well as other immune cells, can be detected in peripheral blood and skin lesions during the progressive stages. The immunological mechanisms underlying vitiligo are assumed to be governed by cellular immunity with involvement of innate and adaptive immunity (Rashighi and Harris 2017). Regarding innate immunity, HSP70 has attracted considerable attention. While in acquired immunity, CD8+ CTLs and the respective upstream interferon (IFN)-γ pathway have been investigated, which resulted in the development of immunotherapy compounds targeting this pathway, such as Janus kinase (JAK) inhibitors (Iannella et al. 2016; Boniface et al. 2018; Wang et al. 2019).

Fructus Lycii is the dried ripe fruit of *Lycium barbarum* L. (Solanaceae). The fruit contains sugars, amino acids, trace elements, alkaloids and other components, among which LBP is the most important pharmacological compound with strong antioxidant effects *in vitro* and *in vivo* (Zhang et al. 2013; Zhao et al. 2015). Our previous studies demonstrated that LBP activated the Nrf2/ARE signalling pathway in a UV-induced photodamage keratinocyte (KC) model (Li, Li, et al. 2017), upregulated the levels of phase-II detoxifying enzymes and antioxidant enzymes (Li, Peng, et al. 2017) and upregulated H_2_O_2_-induced autophagy protein expression in melanocytes. Moreover, LBP induced autophagy and promoted proliferation of PIG1 cells through activation and upregulation of the Nrf2/p62 pathway. Application of monobenzone can be used to induce animal models of skin disease with high similarity to human vitiligo (Van den Boorn et al. 2011; Zhu et al. 2013), and the present research investigates the effect and mechanism of LBP on monobenzone-induced vitiligo in mice.

## Materials and methods

### Reagents

The following reagents and materials were used: TAC and monobenzone (Cat. No. 1642802 and 1445506, respectively; Sigma-Aldrich, China), specific antibodies against chemokine (C-X-C motif) ligand CXCL9, CXCL10, CXCR3 and HSP70 (Cat. No. ab202961, ab271208, ab181013 and ab181606, respectively; Abcam, Cambridge, MA, USA), anti-CD8 monoclonal antibody (Cat. No. 55397; Cell Signaling Technology, Danvers, MA, USA), Trizol and cDNA synthesis kits (Cat. No. 15596018 and 4374967, respectively; Invitrogen, Carlsbad, CA, USA), primers for reverse transcription–quantitative polymerase chain reaction ([RT-qPCR]; Invitrogen, Shanghai, China) and specific antibodies against p-STAT3, STAT3 and GAPDH (Cat. No. 9145 T, 91395 and 5174S, respectively; Cell Signaling Technology).

### Study animals

Female C57BL/6 mice (4–5 weeks old, 10–18 g weight) were obtained from the Experimental Animal Center of Guangdong Province (Guangzhou, China). The mice were housed under standard laboratory conditions and fed a standard diet with free access to water. All experiments were approved by the Animal Welfare and Ethics Branch of the Biomedical Ethics Committee of Guangzhou University of Chinese Medicine (No. 2021054). Vitiligo models were established by topically applying 40% monobenzone (4-benzyloxyphenol; Sigma-Aldrich) in form of a monobenzone cream to four or five-week-old mice for eight weeks, as previously described (Zhu et al. 2013).

### LBP administration

Thirty C57BL/6 mice were randomly assigned to five groups (*n* = 6): as (group I) negative control group, (group II) vitiligo model group induced by monobenzone that were treated with vaseline, (group III) positive control group that were administered with 1 mg/kg TAC orally and ((group IV and group V) treated groups with 0.3 and 0.6 g/kg LBP, respectively. Drug administration began 4 h before the daily application of monobenzone cream for eight weeks.

### Skin depigmentation and histology

Depigmentation was quantified by visual examination after eight weeks (Mosenson et al. 2012), and severity of depigmentation was recorded using five categories (0, none; 1, slight; 2, moderate; 3, marked; 4, severe; 5, very severe). Formalin-fixed skin specimens were embedded in paraffin, sectioned (7 μm thickness) and were stained using haematoxylin and eosin.

### Immunofluorescence

Paraffin-embedded sections were deparaffinized using xylene and ethanol, washed three times for 2 min using distilled water and were soaked in phosphate-buffered saline (PBS) for 5 min. Antigen retrieval was performed in a microwave oven for 30 min, after which the sections were washed twice for 2 min using distilled water. Subsequently, the specimens were washed twice for 2 min using PBS and were blocked for 30 min using serum. The sections were then incubated with rabbit anti-CD8+ monoclonal antibody overnight at 4 °C, followed by washing three times for 5 min using PBS, incubation with fluorescein isothiocyanate-conjugated goat anti-mouse antibody for 2 h, washed 3 times with PBS for 5 min each time, confocal laser scanning microscopy was carried out to capture immunofluorescence.

### Immunochemical analysis

For dewaxing, the sections were heated to 55 °C for 30 min, washed twice for 15 min, rehydrated in ethanol for 15 min, placed in water at 95 °C for 5 min for antigen unmasking and were treated with 3% H_2_O_2_ for 30 min. The sections were then incubated with anti-CXCL9, CXCL10, CXCR3 and HSP70 antibodies at 4 °C overnight, followed by washing in PBS and incubation with secondary antibody at 37 °C for 30 min. 3′3-diaminobenzidine tetrachloride was added to assess positive expression. Negative control sections were incubated with PBS instead of primary antibody. Expression of CXCL9, CXCL10, CXCR3 and HSP70 was quantified using Image-Pro Plus software (Media Cybernetics, Rockville, MD, USA).

### RT-qPCR

Total RNA was extracted from the samples using Trizol (Invitrogen), and RNA concentrations were measured using an ultraviolet spectrophotometer (Beckman Coulter, Brea, CA, USA). RNA was reverse-transcribed to cDNA using a PrimeScript RT Reagent Kit (Takara, China) according to the manufacturer’s instructions and using the following thermocycling protocol: denaturation at 95 °C for 15 s, followed by 35 cycles of denaturation at 95 °C for 5 s and annealing at 61 °C for 15 s. SYBR Premix Ex TaqTM II (Takara) and a quantitative PCR instrument (ViiA7; Thermo Fisher Scientific, Waltham, MA, USA) were used for quantitative analysis. Primer sequences are shown in Table 1. Relative mRNA quantities were determined using the 2^-ΔΔCt^ method, and data were normalized using the GAPDH housekeeping gene.

**Table 1.** Sequence of primers.

Name	Forward	Reverse
IL-8	5′-TCGAGACCATTTACTGCAACAG-3′	5′-CATTGCCGGTGGAAATTCCTT-3′
IL-6	5′-GAGGATACCACTCCCAACAGACC-3′	5′-AAGTGCATCATCGTTGTTCATACA-3′
TNF-α	5′-GGGTGTTCATCCATTCTCTACC-3′	5′-GTCCCAG-CATCTTGTGTTTC-3′
IFN-γ	5′-GCCACGGCACAGTCATTGA-3′	5′-TGCTGATGGCCTGATTGTCTT-3′
IL-1β	5′-GAAATGCCACCTTTTGACAGTG-3′	5′-TGGATGCTCTCATCAGGACAG-3′
GAPDH	5′-AAGAGGGATGCTGCCCTTAC-3′	5′-TACGGCCAAATCCGTTCACA-3′

### Western blotting analysis

Cells were lysed using lysis buffer, followed by centrifugation at 15,000 rpm and 4 °C for 15 min to separate total protein. Total cellular protein concentrations were quantified using a BCA kit. Proteins were resolved using 12.5% sodium dodecyl sulphate–polyacrylamide gel electrophoresis and were then transferred to polyvinylidene difluoride membranes. The membranes were then blocked using 3% bovine serum albumin at room temperature for 30 min, after which they were incubated with different primary antibodies at 4 °C overnight, followed by incubation with secondary antibodies for 1 h. Proteins were detected using a Bio-Rad Imaging System (Bio-Rad Biosciences, Hercules, CA, USA).

### Statistical analysis

Results are expressed as means ± standard error of the mean. Differences between groups were tested using a one-way ANOVA, followed by Student’s *t*-test to compare each pair of columns. Statistical analyses were performed using SPSS software 19.0, and results were visualized using GraphPad Prism 5.0. Statistical significance is reported at *p* < 0.05. All experiments were performed using at least three replicates.

## Results

### LBP ameliorates monobenzone-induced vitiligo-like skin lesion in mice

**Figure 1.**
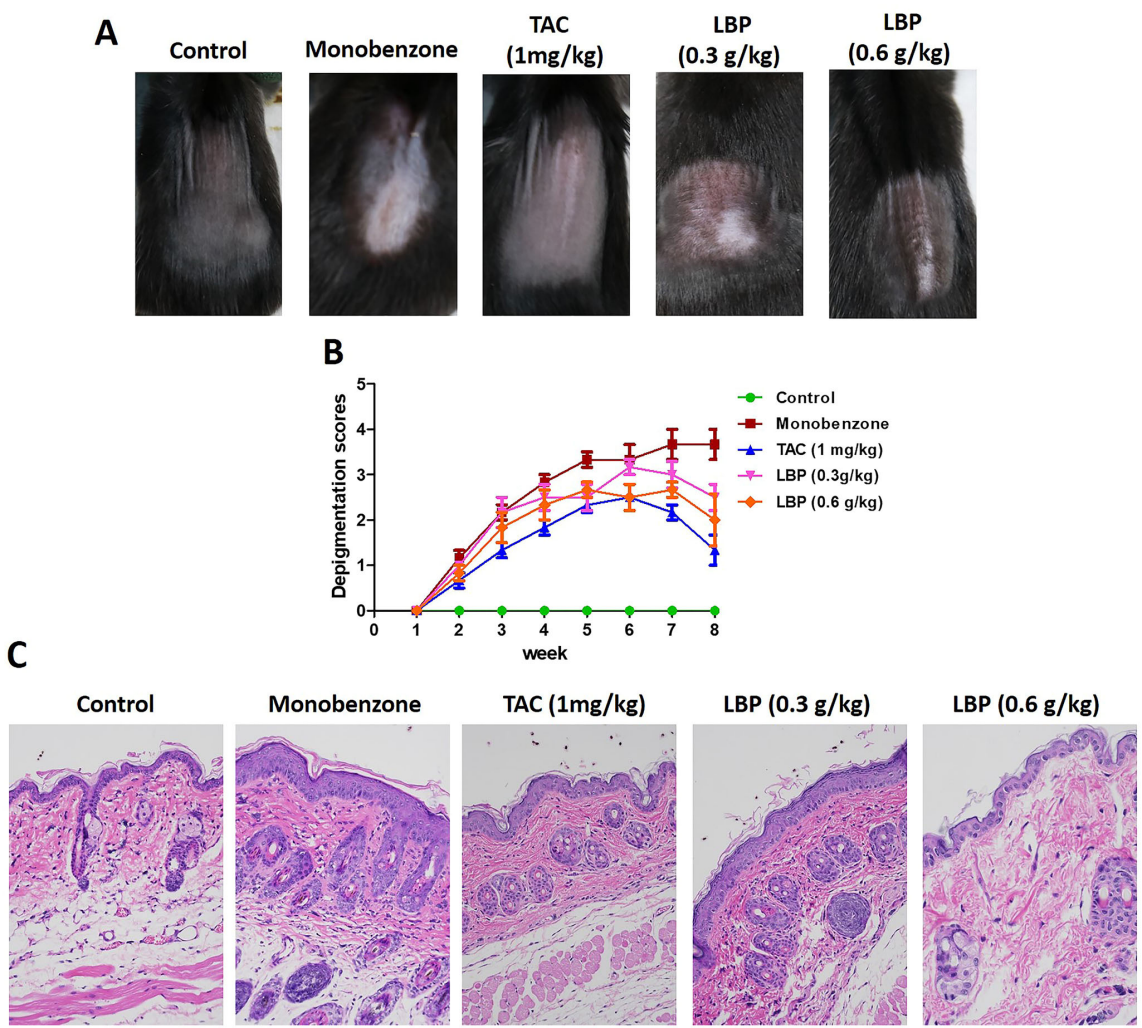
LBP induces a protective effect against monobenzone-induced vitiligo. (A) Photographs of the backs of mice on week 8 after the first monobenzone application. All mice were randomly divided into five groups, and TAC or LBP administration began 4 h before daily monobenzone cream application for eight weeks. (B) Severity of depigmentation during the course of monobenzone -induced vitiligo, as evaluated by the scores on the depigmentation. (C) Histological sections of skin tissue from different groups of mice (haematoxylin and eosin staining; magnification × 200, scale bar = 50 μm).

The efficacy of oral LBP treatment of mice with monobenzone-induced vitiligo-like skin lesions was evaluated. Compared with the monobenzone-only treatment, LBP–treated mice showed significantly reduced depigmentation. The characteristics of monobenzone-induced skin depigmentation were recorded daily, and depigmentation scores were significantly lower in the 0.3 and 0.6 g/kg LBP treatments than in the monobenzone-only group (Figure 1). Analysis of sections stained with haematoxylin and eosin showed distinct epidermal thickening and leukomonocyte infiltration in monobenzone-treated mice (Figure 1), whereas the LBP treatment led to a significant reduction in leukomonocyte abundance. These results indicate that LBP treatment can attenuate vitiligo-like skin lesions induced by monobenzone.

### Effect of LBP on immunofluorescence of CD8+ T cells in the skin of mice

**Figure 2.**
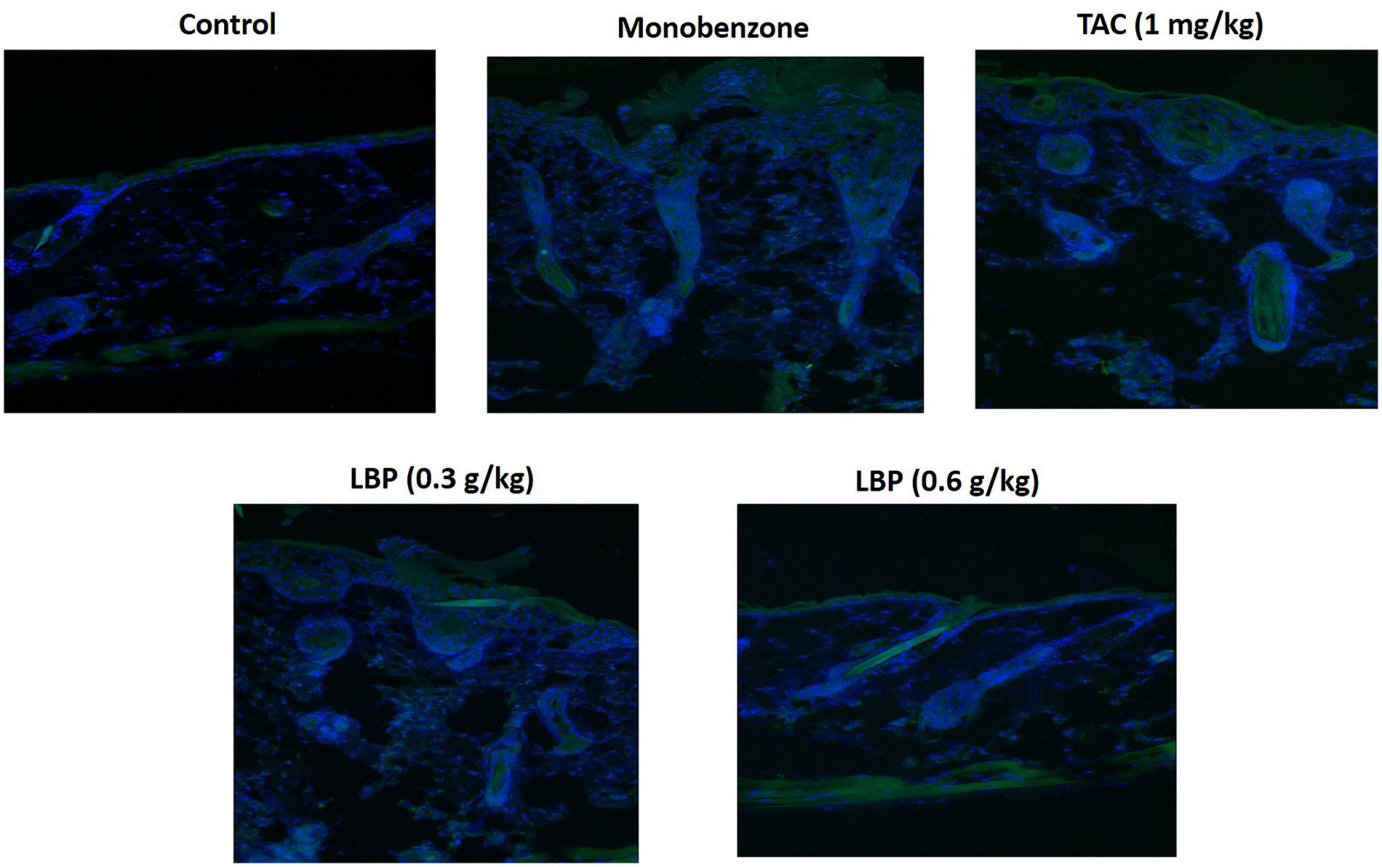
LBP reduced the infiltration of CD8+ T cells in skin lesions of monobenzone induced vitiligo mice. Immunofluorescence Staining for CD8 + T cells in skin sections from different groups of mice. The green fluorescence intensity represents the level of CD8 + expression, and the blue fluorescence is DAPI staining.

To assess whether LBP influences CD8+ T cells in the skin of mice with monobenzone-induced vitiligo, we measured CD8+ T cells expression using immunofluorescence. The LBP treatment showed down-regulated levels of CD8+ T cells in the skin of mice with monobenzone-induced vitiligo (Figure 2).

### LBP inhibits Hsp70-CXCL9/CXCL10 signalling pathway in vitiligo-like skin lesions

**Figure 3.**
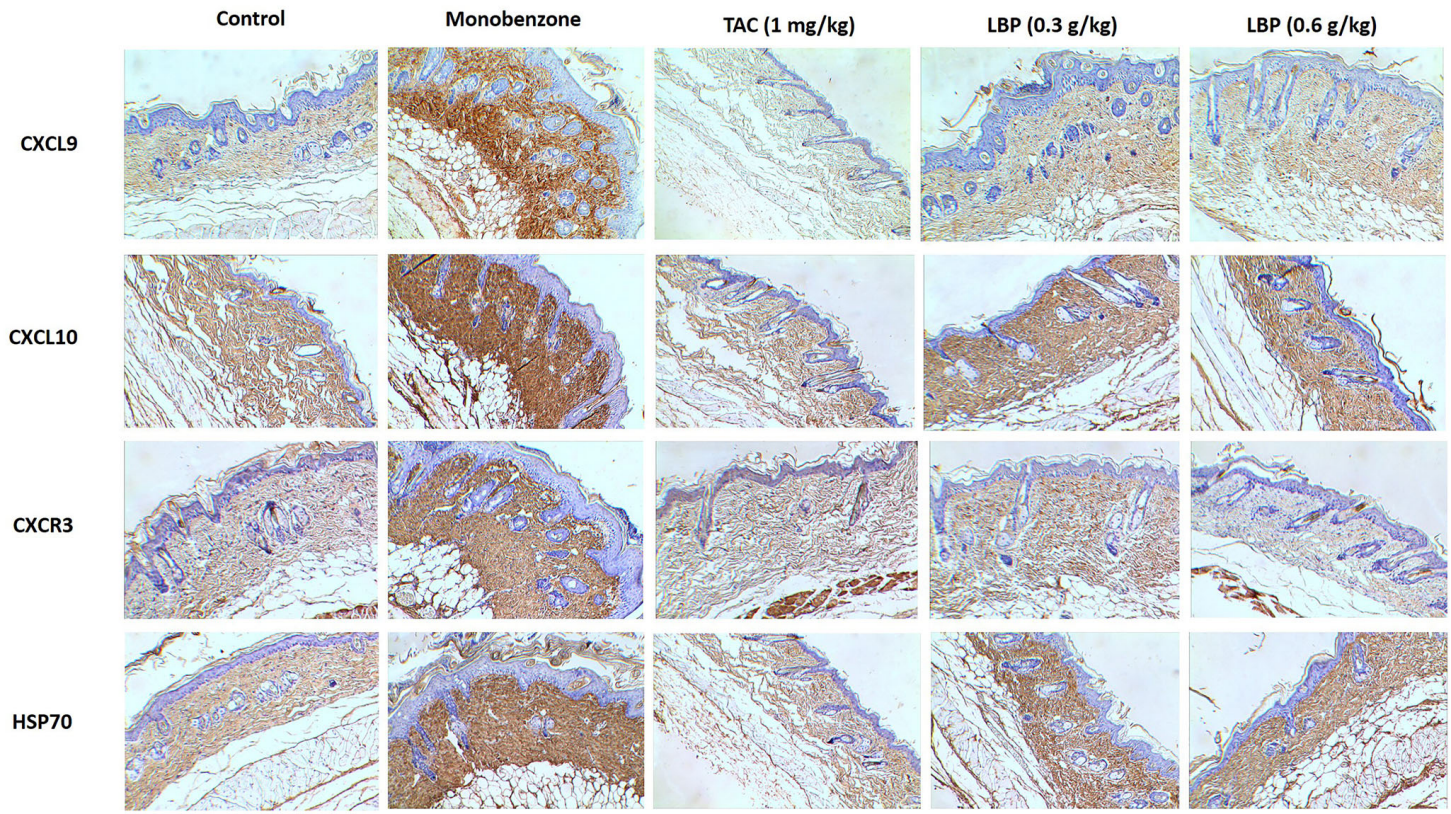
Effects of LBP on the Hsp70-CXCL9/CXCL10 signalling pathway in mice with monobenzone-induced vitiligo. Representative immunochemical photograph of CXCL9, CXCL10, CXCR3 and HSP70 in skin. The depth of brown granules represents the expression of CXCL9, CXCL10, CXCR3 and HSP70, respectively (magnification × 200, scale bar = 50 μm).

The Hsp70-CXCL9/CXCL10 signalling pathway is closely involved in processes of vitiligo (Denman et al. 2008; Mosenson et al. 2012), we therefore investigated the Hsp70-CXCL9/CXCL10 signalling pathway in monobenzone-treated mice. Treatment with LBP inhibited expression of CXCL9, CXCL10, CXCR3 and HSP70, as assessed by immunohistochemistry (Figure 3). Therefore, LBP may inhibit the Hsp70-CXCL9/CXCL10 signalling pathway and thereby prevent induction of CD8+ T cells.

### LBP suppresses mRNA expression of pro-inflammatory cytokines in monobenzone-treated mice

**Figure 4.**
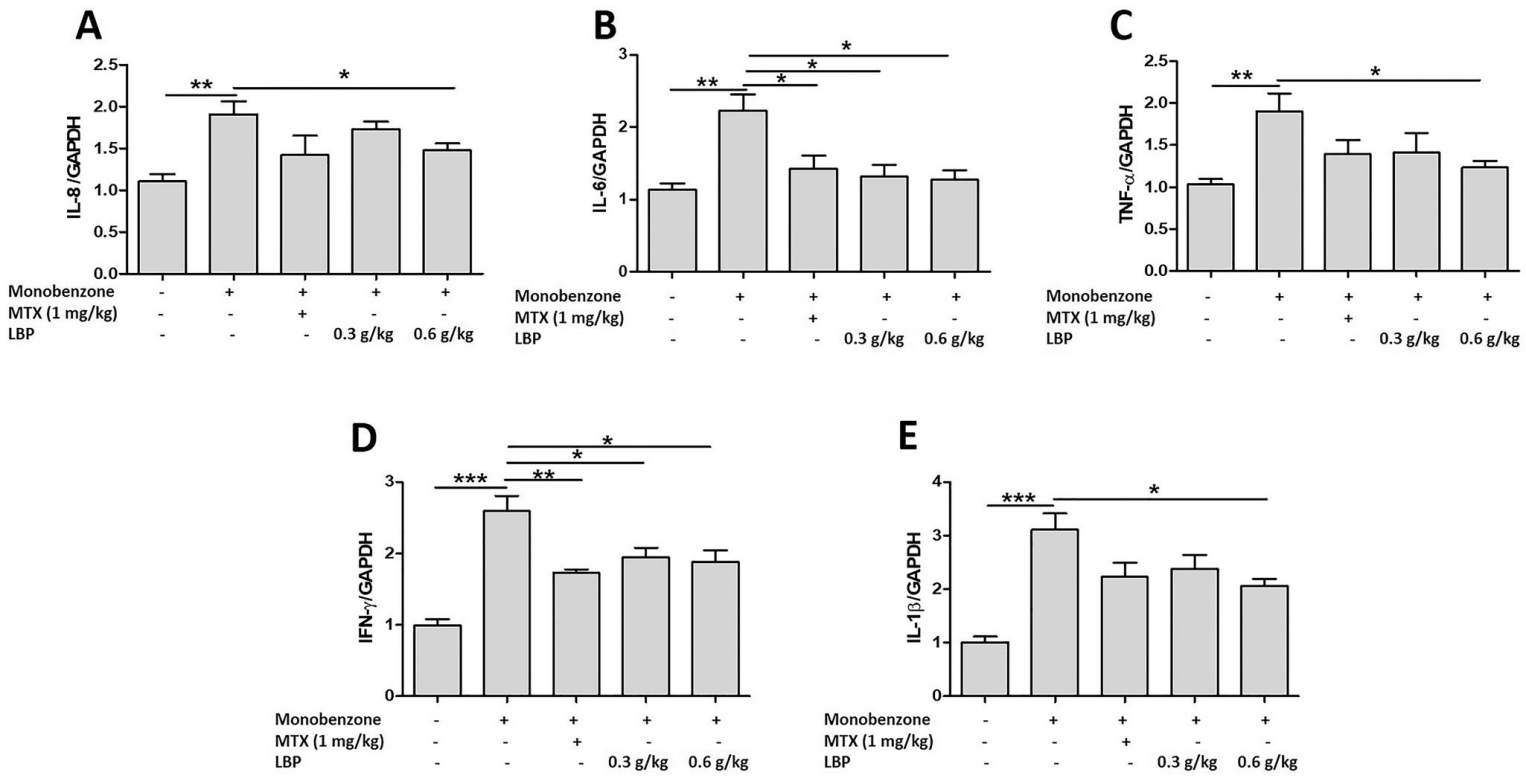
Effects of LBP on inflammatory cytokines of monobenzone-induced vitiligo in mice. Total RNA was isolated from skin tissue, and RT-qPCR was used to investigate the levels of various inflammatory cytokines. Quantification of the amounts of inflammatory cytokines to GAPDH by RT-qPCR. Data are shown as the mean ± standard error of at least three independent experiments (*p < 0.05, **p < 0.01, ***p < 0.001 vs. monobenzone-induced vitiligo group; *n* = 6).

To assess whether LBP influences innate immune responses in the skin of mice with monobenzone-induced vitiligo, we measured IL-8, IL-6, TNF-α, IFN-γ and IL-1β expression by RT-qPCR. The LBP treatment showed down-regulated levels of IL-8, IL-6, TNF-α, IFN-γ and IL-1β (Figure 4).

### LBP inhibits the phosphorylation of STAT3 in vitiligo-like skin lesions

**Figure 5.**
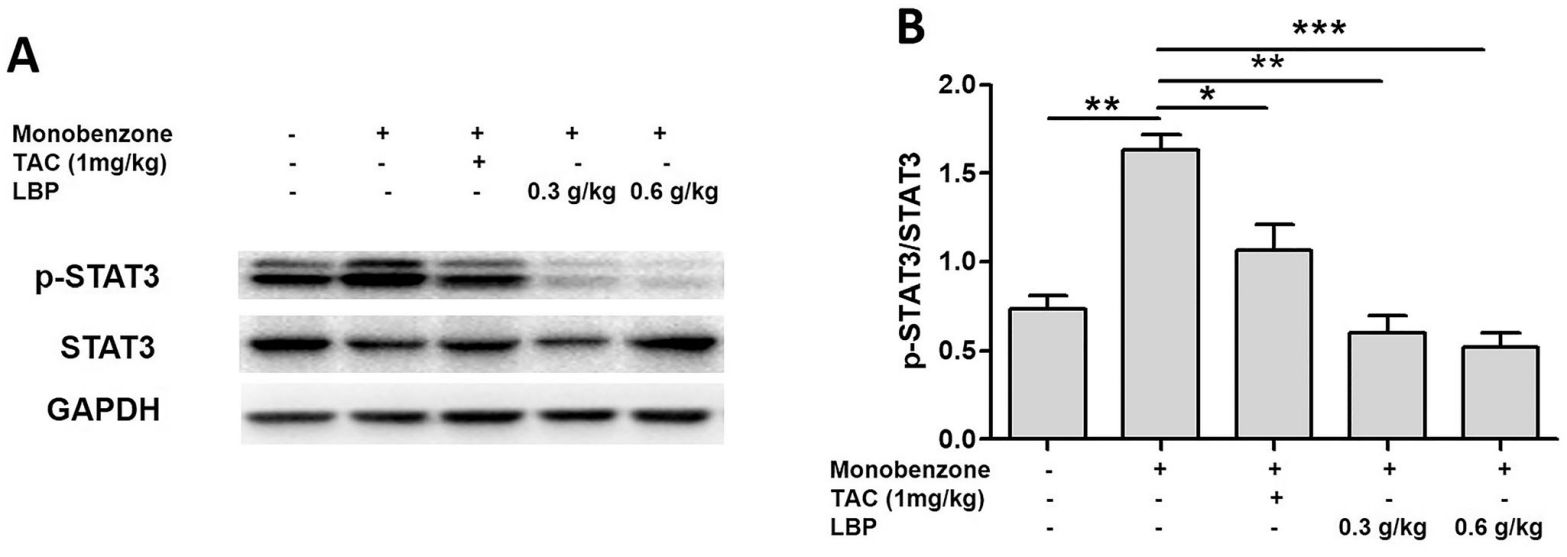
Effects of LBP on the activation of nuclear transcription factor STAT3 in mice with monobenzone-induced vitiligo. (A) Representative western blot of p-STAT3 and STAT3 protein expression in the skin of mice with monobenzone-induced vitiligo. (B) Quantification of the amounts of phospho-STAT3 relative to STAT3 in the skin mice (*p < 0.05, **p < 0.01, ***p < 0.001 vs. monobenzone-induced vitiligo group; *n* = 6).

STAT3 is a crucial nuclear transcription factor upstream of the Hsp70i-CXCL9/CXCL10 signalling pathway (Samaka et al. 2019), we therefore analysed phosphorylation expression of STAT3 and found that the ratio of phospho-STAT3/STAT3 was decreased after the LBP treatment (Figure 5).

## Discussion

Vitiligo is an acquired depigmentation skin disease characterized by the absence of epidermal melanocytes and the occurrence of depigmented white patches on the skin and mucous membranes. Monobenzone is a skin depigmenting agent that acts on melanocyte tyrosinase, induces pigment loss, forms quinone haptens, induces vitiligo-like immune responses through cross-presentation of immature human dendritic cells and autologous T lymphocytes and causes depigmentation in hyperpigmented skin and non-directly exposed areas (Arowojolu et al. 2017). LBP treatment effectively alleviated vitiligo severity in mice treated with monobenzone by reducing the degree of depigmentation.

CD8+ CTLs (cytotoxic T cells) and the activation of the IFN-γ pathway are increased in skin lesions and peripheral blood of vitiligo patients, which is correlated with the severity of disease and compared with normal skin, the number of CTLs at the boundaries of vitiligo skin lesions is significantly increased (Wang et al. 2019). Such CTLs can be activated by melanocyte-specific antigens PMEL (a premelanosome protein) and MART-1 (a melanoma antigen recognized by T cells). In the current study, we found that monophenone-induced depigmentation in mice resulted in loss of CD8+ T infiltration in the lesion area, similar to lesions in human vitiligo. LBP treatment showed significant effects on monobenzone-stimulated histopathological changes in skin by reducing inflammatory infiltration, especially with regard to CD8 + T cell infiltration.

Chemokines are a class of small secreted proteins with similar structures and relative molecular weights of 8000–10,000. These compounds can mediate migration of target cells to effector sites by binding to their receptors, and they are key signal sources for regulating specific migrations of human immune cells, as well as for tissues and organs. When vitiligo occurs, CTLs undergo chemotaxis induced by the corresponding chemokines and migrate to the epidermis to exert specific cytotoxic effect on melanocytes. It was shown that CXCR3 knockout significantly reduced the severity of vitiligo lesions in mice, confirming that migration of vitiligo melanocyte-specific CD8+ T lymphocytes to the epidermis depends on chemokine receptor CXCR3 (Gregg et al. 2010). CXCL9 and CXCL10 in vitiligo lesions target chemotactic CD8+ T lymphocytes to migrate towards the epidermis by binding to CXCR3 (Strassner et al. 2017). With elevated levels of chemokines CXCL9 and CXCL10 in the epidermis, CTLs undergo chemotaxis induced by chemokines and migrate from the vasculature to the epidermis, initiating specific cytotoxic effects by binding specifically to melanocytes and thus leading to melanocyte destruction and occurrence of white spots (Yang et al. 2018). In line with these observations, our results showed highly increased expression of CXCL9, CXCL10, CXCR3 and HSP70 in mice treated with monobenzone. However, LBP treatment inhibited the activation of Hsp70-CXCL9/CXCL10 signalling pathway.

During vitiligo, the innate immune response is abnormally active in the lesions. Natural killer cells (NKs) accumulate and produce a large number of inflammatory proteins and cytokines such as heat shock proteins (HSPs), IL-1β, IL-6 and IL-8 (Henning et al. 2018). Among HSP molecules, inducible HSP70 (HSP70i) has a unique function as it can bind to melanosomal proteins or peptides in melanocytes to assist with protein folding, transport and loading of major histocompatibility complexes I and II (Mosenson et al. 2014). It was shown that IFN-γ produced by T lymphocytes in proximity of vitiligo skin lesions can promote the production of HSP70i, thereby producing positive feedback on Hsp70i-CTL-IFNγ-HSP70i (Xie et al. 2016). A different study found that HSP70i can promote the production of CXCL9 and CXCL10 by KCs through promoting the production of IFN-α by plasmacytoid dendritic cells to induce chemotaxis in CTLs, resulting in formation of the Hsp70i-pDCs-IFN-α-CXCL9/CXCL10-CTL cascade axis (Jacquemin et al. 2017). In the present study, we observed increased levels of IL-8, IL-6, TNF-α, IFN-γ and IL-1β in the skin of monobenzone-induced vitiligo mice, and LBP significantly inhibited secretion of IL-8, IL-6, TNF-α, IFN-γ and IL-1β.

IFN-r-JAK/STAT is an upstream regulatory pathway of CTL-CXCL9/CXCL10. IFN-γ activates JAK in KCs by binding to receptors on KCs. JAK is activated after dimerization, causing phosphorylation of downstream STAT. Phosphorylated STAT enters nuclei and induces transcription of CXCL9 and CXCL10 genes to produce chemokines CXCL9 and CXCL10. CTLs in the vasculature undergo chemotaxis induced by CXCL9 and CXCL10, migrate to the epidermal-dermal junction and exert specific cytotoxic effects on melanocytes, thus leading to vitiligo (Harris et al. 2012). Our study showed that LBP can down-regulate the ratio of p-STAT3/STAT3 in skin lesions of monobenzone-treated mice, suggesting that LBP may inhibit the activation of the STAT3-Hsp70-CXCL9/CXCL10 pathway in vitiligo treatment.

## Conclusions

In mice with monobenzone-induced vitiligo, LBP treatment attenuated the symptoms of a vitiligo-like skin disease, inhibited infiltration of CD8+ T cells and reduced activity of the STAT3-Hsp70-CXCL9/CXCL10 signalling pathway. This may be one mechanism by which LBP affects vitiligo *in vivo*. Further investigation of LBP regarding the relationship between CD8+ T cells and the STAT3-Hsp70-CXCL9/CXCL10 pathway *in vivo* and *in vitro* is needed.
